# Supplementary material for: Bempedoic Acid Restores Liver H2S Production in a Female Sprague-Dawley Rat Dietary Model of Non-Alcoholic Fatty Liver
Source: Int J Mol Sci. 2022 Dec 28;24(1):473. doi: 10.3390/ijms24010473 (PMC9820553; doi:10.3390/ijms24010473)
Supplement: Supplementary file 1 [file ijms-24-00473-s001.zip › ijms-2097612-supplementary.pdf]

Table S1: Primers used for RT-PCR

Gen	*GenBank™ nº*	*Primer sequences*	*PCR product*
Abcg5	NM_053754.2	Forward 5’-CGCAGGAACCGCATTGTAA-3’<br>Reverse 5’-TGTCGAAGTGGTGGAAGAGCT-3’	68 bp
β-Actin	NM_031144.3	Forward 5’-GGCCCGAGTTGATTGACTAT-3’<br>Reverse 5’-CATCCAGCATCTTTGGAATG-3’	111bp
Cbs	NM_012522.2	Forward 5’-GTTTGGTGTTGTCACCGCCATTGA-3’<br>Reverse 5’-CCCTCCTAACGCTCAGCTCCCTAT-3’	206 bp
Cse	NM_017074.2	Forward 5’-GTATGGAGGCACCAACAGGT-3’<br>Reverse 5’-GTTGGGTTTGTGGGTGTTTC-3’	150 bp
Cyp7a1	NM_012942.2	Forward 5’-TACCTGCAAACTGATGGGGAA-3’<br>Reverse 5’-GCTTCTGTGTCCAAATGCCTTC-3’	139 bp
Cyp27a1	NM_178847.3	Forward 5’-GGGGTAGACACGACATCCAA-3’<br>Reverse 5’-AGCTTTTAGCAGGGGCATGT-3’	159 bp
Cyp8b1	NM_031241.2	Forward 5’-TTCCTCAACCCTGATGGCAC-3’<br>Reverse 5’-AGGGCATGTTGTAGTGGTGG-3’	76 bp
Mafg	NM_022386.3	Forward 5’-GGCTCCCGCTTCACCTTTA-3’<br>Reverse 5’-GTGTGAGTGCCTGCTCACT-3’	83 bp
3mst	NM_138843.2	Forward 5’-TTCATCAAGACCCACGAGGATA-3’<br>Reverse 5’-TTCAGTGAATGGGATGTTTACTGAG-3’	162 bp
Pgc1a	NM_031347.1	Forward 5’-CAGAACAAACCCTGCCATTGTT-3’<br>Reverse 5’-GCTTTTGCTGTTGACAAATGCT-3’	77 bp
Rps29	NM_012876.1	Forward 5’-TGAAGGCAAGATGGGTCACCAGCAGC-3’<br>Reverse 5’-CAGGGTAGACAGTTGGTTTCATTGGG-3’	236 bp
Shp	NM_057133.1	Forward 5’-TGATGGCTCCCAAAACCTCC-3’<br>Reverse 5’-AGGAATTCTGCCCTGAAGCA-3’	89 bp
Slc6a6	NM_017206.2	Forward 5’-CCCTGACCTACAACAAAGTCTACCG-3’<br>Reverse 5’-GTTCATGAGGGTTGCTCTGGAGTG-3’	233 bp

Table S2. Antibodies used in western blot analysis.

Antibody	Reference Number
**CYP7A1 (E-10)**	Santa Cruz Biotechnology (sc-518007)
**FXR (H-130)**	Santa Cruz Biotechnology (sc-13063)
**mTOR (7C10)**	Cell Signaling Technology (#2983)
**$P^{Ser2481}$mTOR**	Millipore (#09-343)
**PGC-1α**	Cayman (101707)
**P^{Ser571}-PGC1 α**	R&Dsystems (AF6650)
**S6K1**	Cell Signaling Technology (#9202)
**P^{Thr389}S6K1**	Cell Signaling Technology (#9205)
**TBP**	Sigma Aldrich (SAB4502926)
**VINCULIN (7F9)**	Santa Cruz Biotechnology (sc-73614)
